# HDAC7 Scaffolds GSK3β‐Tau Interaction to Drive GSK3β Activation and Tau Pathology

**DOI:** 10.1111/acel.70830

**Published:** 2026-11-10

**Authors:** Shifeng Xiao, Lijuan Wu, Wenfei Qiao, Ruizhu Yue, Xing Guo, Chengjia Li, Yingling Tang, Jinwang Ye

**Affiliations:** ^1^ Brain Disease and Big Data Research Institute, College of Life Sciences and Oceanography Shenzhen University Shenzhen Guangdong China; ^2^ Shenzhen‐Hong Kong Institute of Brain Science‐Shenzhen Fundamental Research Institutions Shenzhen China

**Keywords:** acetylation, Alzheimer's disease, GSK3β, HDAC7, phosphorylation, scaffolding activity, tau

## Abstract

Intraneuronal accumulation of hyperphosphorylated tau is a pathological hallmark of Alzheimer's disease (AD) and related tauopathies. Although dysregulation of both histone deacetylases (HDACs) and glycogen synthase kinase 3β (GSK‐3β) has been implicated in tau pathogenesis, whether and how they functionally interact to drive tau pathology remains unclear. Here, we identify HDAC7 as a critical scaffold that promotes GSK‐3β‐tau complex formation, leading to GSK‐3β activation and tau hyperphosphorylation in a tauopathy mouse model. HDAC7 is selectively upregulated in human tau‐bearing neurons. Neuronal overexpression of HDAC7 exacerbates tau hyperphosphorylation and cognitive deficits, accompanied by increased GSK‐3β kinase activity and protein levels both in vivo and in vitro. Mechanistically, HDAC7 scaffolds the direct interaction between GSK‐3β and tau independently of its deacetylase activity. Formation of the HDAC7‐GSK3β‐tau complex enables tau to promote GSK‐3β acetylation at lysine 15, which in turn stabilizes GSK‐3β protein and enhances its kinase activity. Conversely, neuronal deletion of HDAC7 reduces GSK‐3β protein levels and kinase activity, attenuates tau hyperphosphorylation, restores synaptic integrity, and rescues cognitive deficits in hTau‐expressing mice. Together, our findings propose a novel mechanism by which HDAC7 scaffolds GSK3β‐tau interaction to drive sustained GSK‐3β activation and tau pathology, highlighting reducing HDAC7 as a potential therapeutic strategy for AD and other tauopathies.

## Graphical abstract

HDAC7, through its N‐terminal protein‐interaction domain, scaffolds GSK3β and tau to promote GSK3β K15 acetylation, kinase activation, and tau hyperphosphorylation, ultimately driving cognitive decline. Targeting HDAC7 scaffolding offers a potential therapeutic strategy for tauopathies.

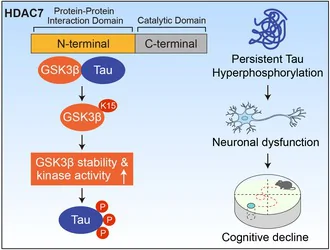

## 1 Introduction

The microtubule‐associated protein tau is integral to the pathogenesis of Alzheimer's disease (AD) and several other neurodegenerative diseases, such as progressive supranuclear palsy (PSP), Pick's disease, corticobasal degeneration and frontotemporal dementia (Parra Bravo et al. 2024). Within neurons, tau is predominantly localized in axons, where it functions to stabilize neuronal microtubules and facilitate axonal transport under normal physiological conditions. However, in pathological states, tau undergoes aberrant post‐translational modifications, particularly hyperphosphorylation, leading to its misfolding, aggregation, and the formation of neurofibrillary tangles (NFTs) (Islam et al. 2025; Ye et al. 2024). The degree of NFTs is closely associated with neurodegeneration and cognitive decline in AD patients (Nelson et al. 2012). Nevertheless, the mechanisms underlying the persistent hyperphosphorylation and gradual aggregation of tau proteins during the progression of AD remain unclear.

Phosphorylation is the most abundant post‐translational modification observed in tau, with more than 40 phosphorylation sites linked to the pathological features characteristic of AD (Wesseling et al. 2020). The hyperphosphorylation of tau in AD is hypothesized to result from disruptions in the activity of tau‐associated protein kinases and phosphatases, with glycogen synthase kinase 3β (GSK3β) being the most extensively studied kinase associated with tau pathology (Qian et al. 2010; Wang et al. 2015). Analysis of postmortem AD brain samples indicates that GSK3β colocalizes with hyperphosphorylated tau aggregates in neurons, and its activity is elevated in the brains of AD patients and mouse models (Leroy et al. 2002). In vitro evidence demonstrated that GSK3β directly interacts with tau and catalyzes the aggregation of tau protein into AD‐like filaments, comparable to the paired helical filaments isolated from AD patients (Chakraborty et al. 2024). Moreover, although GSK3β is generally considered an upstream factor relative to tau pathology, recent studies have revealed that tau protein can in turn activate GSK3β, through directly acetylating GSK3β using its acetyltransferase activity (Q. Zhou et al. 2022). These findings suggest the intriguing possibility that GSK3β and tau may form a vicious cycle that exacerbates tau pathology and AD neurodegeneration. However, the regulatory mechanisms underlying the establishment of this vicious cycle during tau pathology are yet not well understood.

Among the 11 zinc‐dependent classical histone deacetylases (HDACs) expressed in the central nervous system, HDAC4, 5, 7, and 9 are categorized as class IIa HDAC enzymes. These enzymes are often considered deacetylase‐inactive within the nucleus, due to the substitution of a critical active site tyrosine with histidine in the active pocket (Lahm et al. 2007). They are capable of shuttling between the nucleus and cytoplasm, enabling them to deacetylate lysine residues of non‐histone proteins in the cytosol and to function as scaffolds or modifiers of non‐nuclear substrates (Wang et al. 2023). Dysregulation or overexpression of class IIa HDACs has been implicated in the pathogenesis of various neurological diseases, through both enzymatic and non‐enzymatic mechanisms. For instance, HDAC7 has been reported to exhibit deacetylate activity on PTEN‐induced putative kinase 1 and inhibitory κB kinase in astrocytes, which correlates with anxiety and depression‐like behaviors in mice (Ye et al. 2022; Yue et al. 2025). HDAC4 associates with huntingtin, and downregulation of HDAC4 transmits beneficial effects to Huntington's disease‐related neurodegenerative phenotypes involving mechanisms not related to its enzymatic activity (Mielcarek et al. 2013). Additionally, HDAC7 demonstrates a deacetylase‐independent proinflammatory role in macrophages, where a reduction in proinflammatory cytokines is attributed to HDAC7 degradation rather than inhibition (Kadier et al. 2024). Our previous study has established a functional crosstalk between astrocytic HDAC7 and neuronal tau pathology in a tauopathy mouse model (Ye et al. 2025). Given that tau forms hyperphosphorylated aggregates primarily within neurons, elucidating the role of neuronal class IIa HDACs in tau pathogenesis could offer new insights into the application of HDAC inhibitors for the treatment of AD and related tauopathies.

In the present study, we observed an upregulation of HDAC7 expression in the cytosol of human tau‐expressing neurons in the brains of an AAV‐mediated tauopathy mouse model. The Overexpression of HDAC7 was found to accelerate tau phosphorylation and exacerbate cognitive impairments in mice, accompanied with an increase in both the protein level and kinase activity of GSK3β. We identified a novel non‐enzymatic function of HDAC7 as a scaffold for GSK3β‐tau interaction, whereby tau acetylates GSK3β at K15, resulting in its stabilization and overactivation. Neuronal deletion of HDAC7 attenuated GSK3β upregulation and overactivation, reduced tau pathological phosphorylation, and ameliorated synaptic and cognitive deficits the in tauopathy mouse model.

## 2 Results

### 2.1 HDAC7 Is Upregulated in the Cytosol of Human Tau‐Expressing Neurons In Vivo and In Vitro

Observations from human samples indicate that hyperphosphorylated tau aggregates are predominantly located intraneuronal in the pathogenesis of AD. To model tau accumulation observed in human AD, we engineered an adeno‐associated virus (AAV) expressing full length human tau (hTau, 2N4R) under the neuron‐specific promoter hSyn. Subsequently, we microinjected AAV‐hSyn‐hTau‐GFP or control AAV‐hSyn‐GFP into the hippocampal CA3 region of 2‐month‐old wild‐type (WT) mice, a region particularly susceptible to the accumulation of hyperphosphorylated tau under various treatments (Li et al. 2016) (Figure 1A). One month post‐injection, robust expression of the neuron‐specific AAV in the hippocampal CA3 subset was confirmed using confocal microscopy (Figure 1B). We have previously showed that this hTau‐overexpression mouse model can induce tau hyperphosphorylation and the formation of tau oligomers and fibrils, which are closely associated with the early stages of AD tau pathology (Ye, Yin, Liu, et al. 2020; Ye, Yin, Yin, et al. 2020).

**FIGURE 1:**
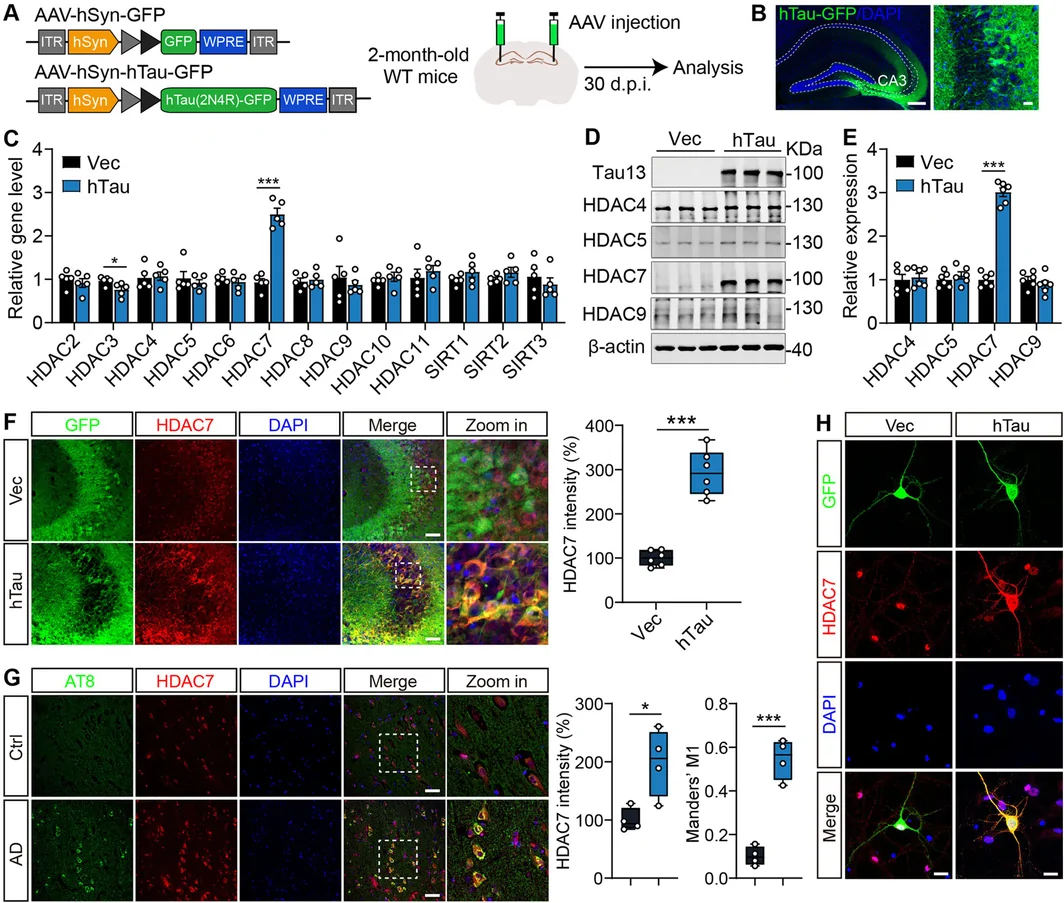
HDAC7 is upregulated in tau‐accumulated neurons. (A) Schematic diagram illustrating the in vivo experimental timeline. AAV‐hSyn‐hTau‐GFP or control AAV‐hSyn‐GFP was stereotaxically infused into the CA3 region of WT mice. (B) Representative immunofluorescence imaging of hippocampus and the enlarged CA3 subset after infusion of AAV‐hTau‐GFP for 4 weeks. Scale bars: 200 μm (Left) and 20 μm (Right). (C) mRNA levels of HDAC2‐11 and SIRT1‐3 analyzed by RT‐qPCR in the hippocampal CA3 of mice after AAV injection. *n* = 5 mice per group. (D, E) Representative Western blot analysis and quantification of class IIa HDACs (HDAC4, 5, 7, and 9) in the hippocampal CA3 extracts from mice after AAV injection. *n* = 6 mice per group. (F) Immunofluorescence staining analysis of HDAC7 in the hippocampal CA3 region of WT mice after AAV injection. *n* = 6 mice per group. Scale bar: 80 μm. (G) Immunofluorescence staining analysis of HDAC7 in the cortical brain sections of AD patients and control individuals, with quantification of the Manders' M1 coefficient (HDAC7 overlapped with AT8). *n* = 4 per group. Scale bar: 80 μm. (H) Representative immunostaining images showing the expression of HDAC7 in primary cultured neurons transfected with hTau‐GFP or GFP plasmids. Scale bar: 10 μm. Statistical significance was determined by unpaired Student's *t*‐test. Data are shown as mean ± SEM, *p < 0.05, **p < 0.01, ***p < 0.001.

To explore the role of HDACs in tau pathology, we examined the mRNA levels of various HDACs in the hippocampal CA3 tissue samples dissected from mice injected with hTau or control GFP. RT‐qPCR analysis showed that, compared with the control mice, the mRNA level of HDAC7 was uniquely upregulated in the hTau‐expressing mice (Figure 1C). HDAC7 is a member of the class IIa HDAC subfamily, sharing structural and functional similarities with other family members, including HDAC4, HDAC5, and HDAC9. Consequently, we assessed the protein levels of class IIa HDACs in the hippocampal CA3 tissue lysates. Western blot analysis showed that HDAC7 protein level was significantly upregulated in the hTau‐expressing mice, with other class IIa HDACs remaining unchanged (Figure 1D,E), consistent with the mRNA findings. Through immunofluorescence staining, we observed that the increased expression of HDAC7 was localized in the cytoplasm of hTau‐positive neurons within the hippocampal CA3 region (Figure 1F). To ascertain whether this phenomenon is observable in other tauopathy mouse models, we conducted co‐staining of HDAC7 and human tau in an alternative hTau transgenic mouse line. Consistently, HDAC7 was specifically upregulated in hTau‐positive neurons in the hippocampus of 13‐month‐old hTau mice (Figure S1A). To further investigate the relevance of HDAC7 dysregulation in human pathology, we examined HDAC7 expression in postmortem cortical brain sections from AD patients and age‐matched controls. Immunofluorescence staining revealed that HDAC7 levels were markedly elevated in AD patients and co‐localized with AT8‐positive tau aggregates (Figure 1G). Moreover, the cytoplasmic enrichment of HDAC7 was also observed in hTau‐overexpressing primary neurons, as confirmed by immunofluorescence staining and nuclear/cytoplasmic fractionation assays (Figure 1H; Figure S1B,C). Taken together, these data suggest that HDAC7 is specifically upregulated in the cytoplasm of tau‐accumulating neurons during tau pathology.

### 2.2 Overexpression of HDAC7 in Neurons Exacerbates Tau Hyperphosphorylation and Aggravates Cognitive Deficits in Mice

To investigate the functional role of neuronal HDAC7 in tau pathology, we employed an AAV‐mediated delivery strategy to overexpress either HDAC7 or a control GFP alongside hTau in the hippocampal CA3 region of 2‐month‐old WT mice (Figure 2A). After 1 month, the expression of hTau‐GFP and HDAC7 in the hippocampal CA3 was verified by immunostaining with HDAC7 (Figure 2B). The cognitive function of mice with neuronal overexpression of HDAC7 and hTau was measured using behavioral tests. The Morris water maze (MWM) behavioral test demonstrated that mice with hTau overexpression exhibited cognitive impairments as compared with the control GFP group (Figure 2C–G). Notably, overexpression of HDAC7 further intensified the cognitive decline observed in the hTau group, as indicated by increased latency to reach the platform during the training phase and probe test (Figure 2C–E), reduced time spent in the platform zone (Figure 2F), and fewer platform crossings during the probe phase (Figure 2G), while swimming speed remained consistent across all groups (Figure 2H). Additionally, the Novel Object Recognition (NOR) test demonstrated that mice with HDAC7 overexpression exhibited a significant reduction in discrimination for a novel object in both hTau‐expressing and control GFP mice, suggesting impaired cognitive function by HDAC7 (Figure 2I).

**FIGURE 2:**
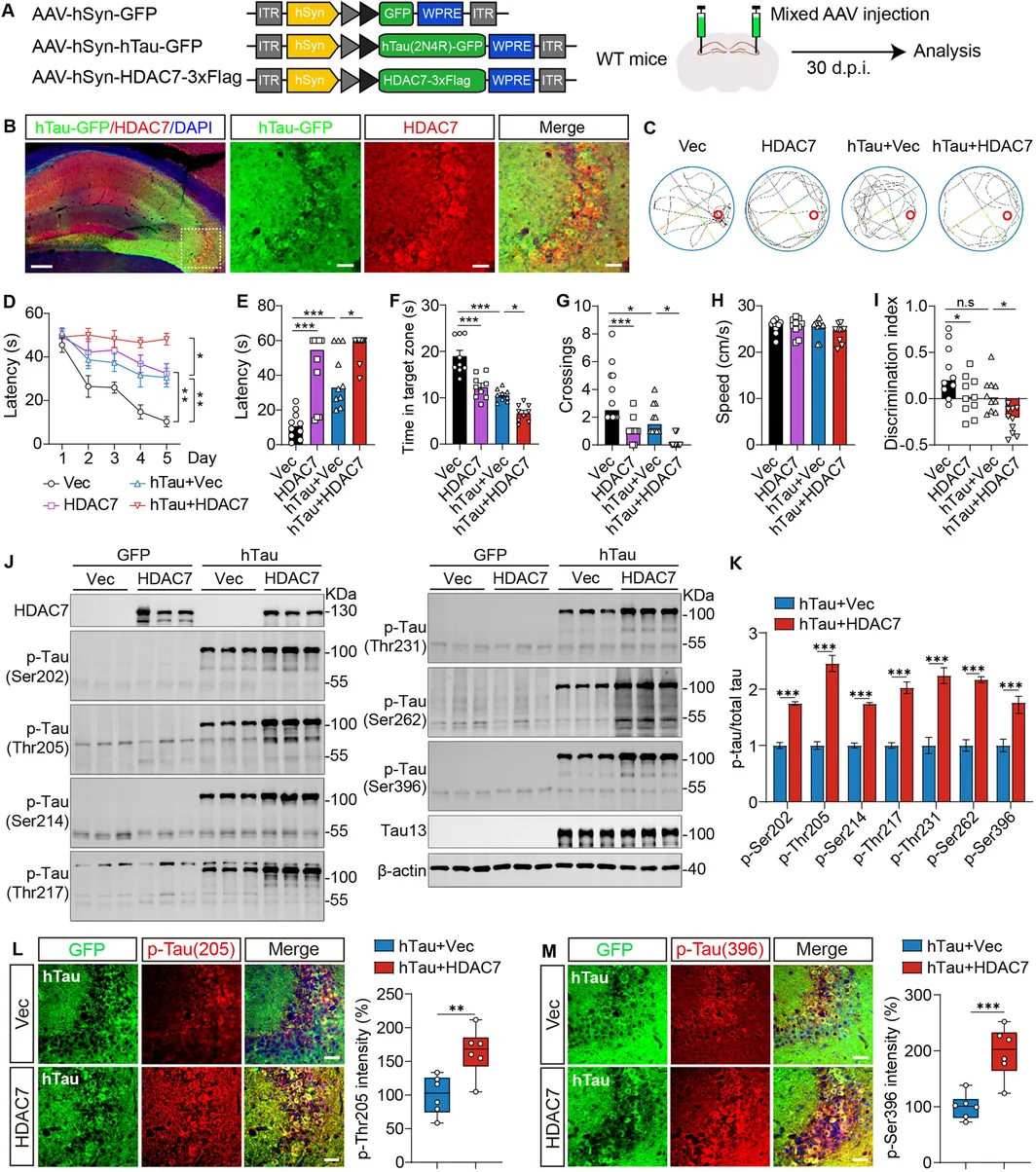
Neuronal overexpression of HDAC7 exacerbates cognitive deficits and tau hyperphosphorylation in hTau‐expressing mice. (A) Schematic diagram illustrating the construction of AAV and the in vivo experimental timeline. (B) Representative immunofluorescence image showing the expression of hTau‐GFP and HDAC7 in hippocampus and the enlarged CA3 region after infusion of AAVs for 4 weeks. Scale bars: 200 μm (Left) and 40 μm (Right). (C–H) Morris water maze (MWM) behavioral analysis of the AAV‐injected mice. (C) Representative travel traces of the mice, (D) escape latency during the training phase, (E) first latency to reach the platform, (F) exploring time in the target zone, (G) number of platform crossings, and (H) swimming speed during the probe test. (I) Discrimination index of mice in the probe trial of Novel object recognition (NOR) behavioral test. For behavioral tests, *n* = 10 mice per group. (J, K) Western blot analysis and quantification of phosphorylated tau (p‐Ser202, p‐Thr205, p‐Ser214, p‐Thr217, p‐Thr231, p‐Ser262 and p‐Ser396), total human tau (Tau13), and HDAC7 in the hippocampal CA3 lysates. *n* = 6 mice per group. (L, M) Immunostaining analysis with quantification of p‐Tau (Thr205) and p‐Tau (Ser396) in the hippocampal CA3 region. *n* = 6 mice per group. Scale bar: 40 μm. Statistical significance was determined by unpaired Student's *t*‐test (K–M) or one‐way or two‐way ANOVA with Tukey's post hoc analysis (D–I). Data are shown as mean ± SEM, *p < 0.05, **p < 0.01, ***p < 0.001, n.s., not significant.

Western blot analysis revealed that HDAC7 overexpression increased hTau phosphorylation levels at multiple disease‐associated sites, including p‐Ser202, p‐Thr205, p‐Ser214, p‐Thr217, and p‐Thr231 in the proline‐rich domain, p‐Ser262 within the microtubule‐binding region, and p‐Ser396 in the C‐terminal region (Figure 2J,K). Furthermore, immunostaining analysis indicated that HDAC7 overexpression significantly increased tau phosphorylation at Thr205 and Ser396 residues in the hippocampal CA3 region of hTau‐expressing mice (Figure 2L,M). Previous studies have established that tau can undergo acetylation, which regulates its pathological accumulation and related cognitive dysfunction (Cohen et al. 2013; Min et al. 2010). However, the levels of acetylated‐tau (ac‐K174) were also increased in the HDAC7‐overexpressing group as compared to the control GFP group, suggesting a deacetylation‐independent mechanism by which HDAC7 influences tau accumulation (Figure S2). Collectively, these findings indicate that neuronal overexpression of HDAC7 enhances tau phosphorylation, which may contribute to exacerbated cognitive impairments in the tauopathy mouse model.

### 2.3 Overexpression of HDAC7 Increases GSK3β Kinase Activity and Protein Level

Tau hyperphosphorylation is attributed to an imbalance in the activity levels or regulation of tau kinases and phosphatases. Given that HDAC7 overexpression markedly aggravates tau phosphorylation while exerting minimal effects on tau acetylation, we investigated the expression levels and activity‐dependent changes of relevant upstream tau kinases and phosphatases. Our results showed that HDAC7 overexpression led to a reduction in p‐GSK3β levels at Ser9 (inactive) and an increase in p‐GSK3β levels at Tyr216 (active) in both the GFP and hTau group (Figure 3A–C), implying enhanced GSK3β activity due to HDAC7. Furthermore, HDAC7 was found to elevate GSK3β protein levels in the brain (Figure 3A,D). The activity of CDK5, another critical kinase involved in tau phosphorylation, was also upregulated by HDAC7 in hTau group mice (Figure 3A,E). The levels of the tyrosine kinase Fyn were consistent across all experimental groups (Figure 3A,F). Furthermore, we assessed protein phosphatase 2A (PP2A), the most active phosphatase for regulating tau phosphorylation at multiple pathological sites (Liu et al. 2005). HDAC7 was observed to reduce the levels of PP2A catalytic subunit in both GFP and hTau group (Figure 3A,G). Immunostaining analysis further demonstrated that HDAC7 markedly increased the immunoreactivity of p‐GSK3β (Tyr216) in the hippocampal CA3 region of hTau‐expressing mice (Figure 3H). Furthermore, GSK3β kinase activity assay showed that GSK3β activity was significantly elevated in HDAC7‐overexpressing mice and further increased upon hTau co‐expression (Figure 3I). Notably, the mRNA levels of GSK3β were consistent in the brain, irrespective of HDAC7 or hTau overexpression (Figure 3J). Collectively, these data suggest that HDAC7 disrupts the regulation of tau upstream kinases and phosphatase, which aggravate tau hyperphosphorylation in mice.

**FIGURE 3:**
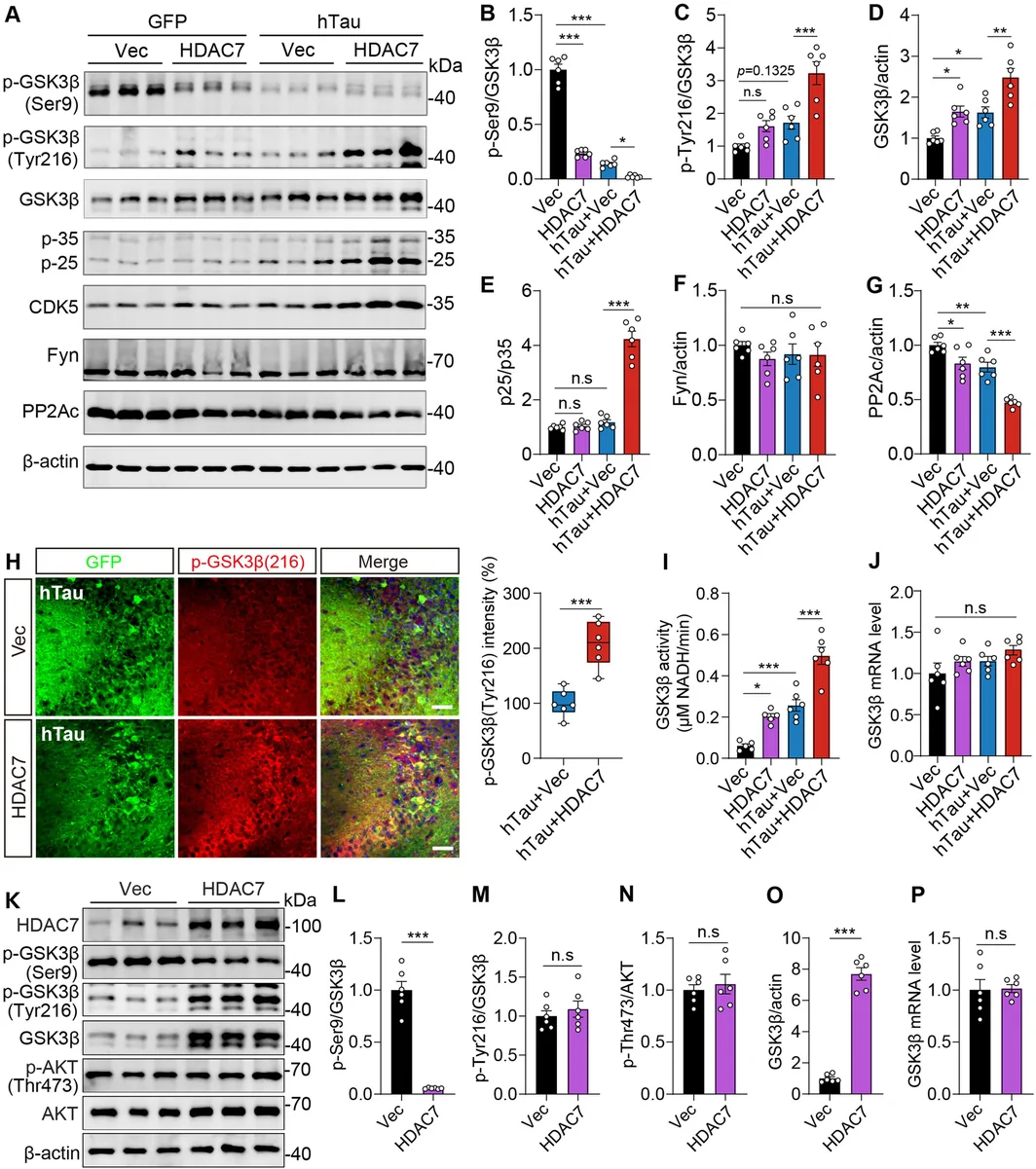
HDAC7 overexpression upregulates GSK3β protein level and enhances its kinase activity independently of AKT signaling. (A) Western blot analysis of p‐GSK3β (Ser9), p‐GSK3β (Tyr216), total GSK3β, p35‐p25, CDK5, Fyn, and PP2Ac in the hippocampal CA3 extracts from mice overexpressed with hTau and HDAC7. (B–G) Quantification of the blots in A: (B) p‐GSK3β (Ser9)/GSK3β, (C) p‐GSK3β (Tyr216)/GSK3β, (D) GSK3β/actin, (E) p25/p35, (F) Fyn/actin, (G) PP2Ac/actin. *n* = 6 mice per group. (H) Immunofluorescence staining analysis with quantification of p‐GSK3β (Tyr216) in the hippocampal CA3 of mice after AAV injection. *n* = 6 mice per group. Scale bar: 40 μm. (I) GSK3β kinase activity measured in the hippocampal CA3 lysates of mice after AAV injection. *n* = 5–6 mice per group. (J) RT‐qPCR analysis showing the mRNA levels of GSK3β in the hippocampal CA3 tissue samples. *n* = 6 mice per group. (K) Western blot analysis of HDAC7, p‐GSK3β (Ser9), p‐GSK3β (Tyr216), total GSK3β, p‐AKT (Thr473), and total AKT in primary cultured neurons overexpressed with HDAC7 or control vector via lentivirus. (L–O) Quantification of the blots in (K): (L) p‐GSK3β (Ser9)/GSK3β, (M) p‐GSK3β (Tyr216)/GSK3β, (N) p‐AKT (Thr473)/AKT, (O) GSK3β/actin. *n* = 6 mice per group. (P) RT‐qPCR analysis showing the mRNA levels of GSK3β in HDAC7‐overexpressing and control neurons. *n* = 6 per group. Statistical significance was determined by unpaired Student's *t*‐test (H, L–P) or one‐way ANOVA with Tukey's post hoc analysis (B–G, I, J). Data are shown as mean ± SEM, *p < 0.05, **p < 0.01, ***p < 0.001, n. s, not significant.

To further elucidate the role of HDAC7 in modulating tau upstream regulators, we assessed their activity and expression levels in primary cultured neurons. In alignment with the observation in vivo, HDAC7 overexpression significantly decreased the levels of p‐GSK3β (Ser9)/GSK3β, although p‐GSK3β (Tyr216)/GSK3β remained unchanged (Figure 3K–M). However, the activity of AKT, the upstream kinase that inhibits GSK3β via direct phosphorylation at the S9 site, remained unchanged following HDAC7 overexpression (Figure 3N). This suggests that the increased activity of GSK3β due to HDAC7 overexpression occurs independently of AKT activation. Notably, the protein expression of total GSK3β was increased in neurons with HDAC7 overexpression (Figure 3K,O), while its mRNA levels remained constant (Figure 3P), indicating a potential impairment in the proteolysis of this kinase. Additionally, the activities of CDK5, Fyn, and PP2A did not show significant changes in HDAC7‐overexpressing cells compared to control cells (Figure S3). Integrating these in vivo and in vitro findings, our data point to GSK3β being the primary regulator of tau hyperphosphorylation mediated by HDAC7.

### 2.4 HDAC7 Stabilizes GSK3β by Facilitating Tau‐Mediated GSK3β K15 Acetylation and Reducing Its Ubiquitination

GSK3β is intricately linked to the pathological phosphorylation of tau, and acetylation has been implicated in the modulation of GSK3β activity and AD tau pathology (Q. Zhou et al. 2022). To determine whether HDAC7 can directly regulate the acetylation status of GSK3β, we conducted co‐immunoprecipitation (IP) in HEK293T cells expressing GSK3β‐3xFlag and HDAC7 or a control vector. The Co‐IP results demonstrated that, although HDAC7 interacts with GSK3β, it does not influence the acetylation level of GSK3β in these cells (Figure S4). Considering that HDAC7 induces a more pronounced upregulation of GSK3β protein levels in the brains of hTau‐expressing mice compared to the control GFP mice (Figure 3A–D), we further assessed the acetylation status of GSK3β in the presence of hTau. Contrary to expectations, HDAC7 overexpression resulted in significantly increased GSK3β acetylation status in the presence of hTau (Figure 4A,B). These data indicate that HDAC7 may indirectly enhance GSK3β acetylation, relying on human tau presence.

**FIGURE 4:**
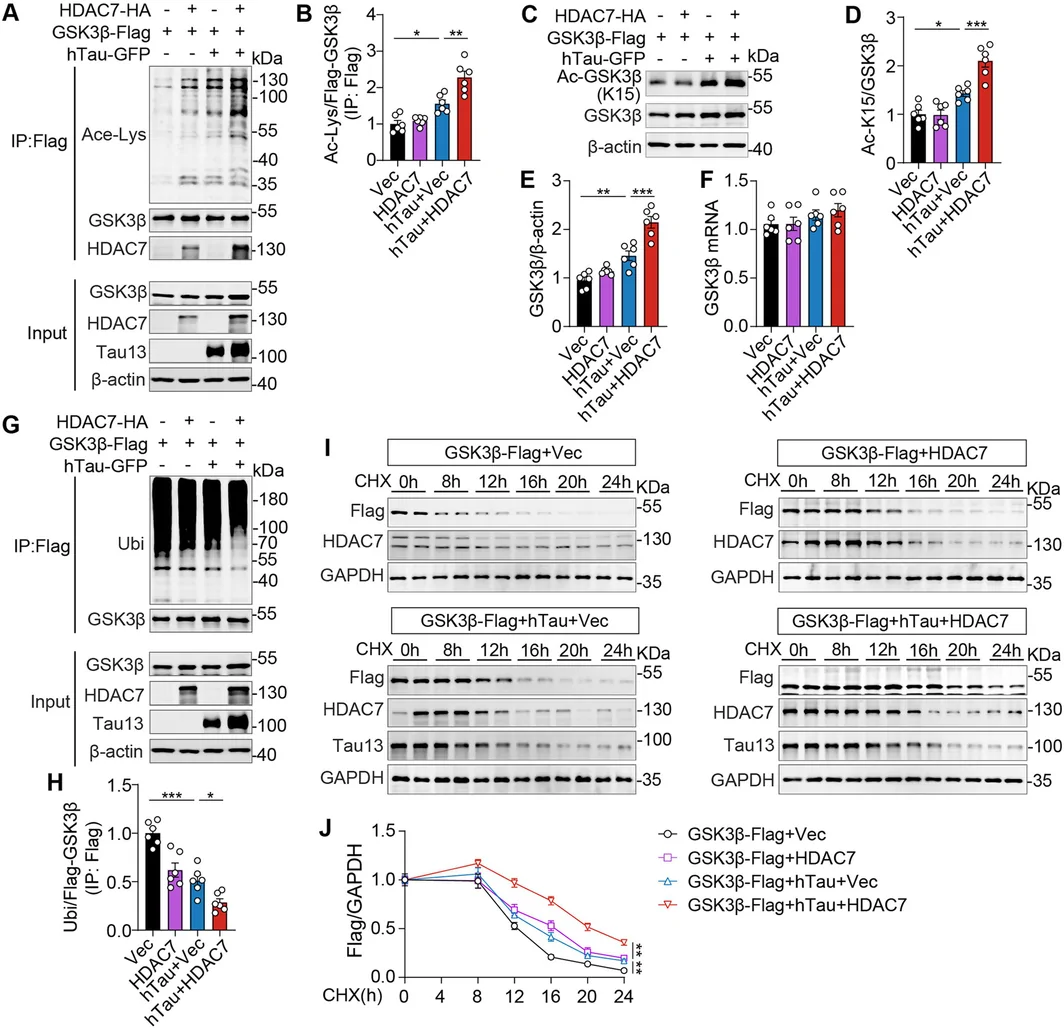
HDAC7 stabilizes GSK3β by promoting tau‐mediated GSK3β K15 acetylation and suppressing its ubiquitination. (A, B) Immunoprecipitation (IP) analysis of GSK3β acetylation levels in HEK293T cells co‐transfected with GSK3β‐Flag, hTau‐GFP, and HDAC7‐HA. *n* = 6 per group. (C–E) Western blot analysis and quantifications of ac‐GSK3β (K15) and total GSK3β in HEK293T cells co‐transfected as indicated. *n* = 6 per group. (F) RT‐qPCR analysis of GSK3β mRNA levels in HEK293T cells. *n* = 6 per group. (G, H) IP analysis of GSK3β ubiquitination levels in HEK293T cells co‐transfected with GSK3β‐Flag, hTau‐GFP, and HDAC7‐HA. *n* = 6 per group. (I, J) Western blot analysis and quantifications of GSK3β‐Flag levels in HEK293T cells co‐transfected as indicated and treated with cycloheximide (CHX) for indicated periods of time. *n* = 6 per group. Statistical significance was determined by one‐way ANOVA with Tukey's post hoc analysis. Data are shown as mean ± SEM, *p < 0.05, **p < 0.01, ***p < 0.001, n.s., not significant.

Previous study has demonstrated that tau protein exhibits acetyltransferase activity, enabling it to acetylate GSK3β at the K15 residue (Cohen et al. 2013) (Q. Zhou et al. 2022). This acetylation enhances GSK3β kinase activity and interferes with its proteolysis. Consistent with these findings, we did observe an upregulation of GSK3β acetylation at K15 by hTau (Figure 4C,D). Notably, our results showed that overexpression of HDAC7 remarkably reinforced hTau‐induced GSK3β K15 acetylation (Figure 4C,D). The identity and specificity of the GSK3β (K15) antibody was verified with dot blot and GSK3β K15R mutation (Figure S5). Furthermore, HDAC7 overexpression was associated with an increase in GSK3β protein levels without affecting its mRNA levels in hTau‐expressing cells (Figure 4C,E,F), suggesting that HDAC7 may enhance GSK3β stability. Analysis of GSK3β immunoprecipitation samples revealed that HDAC7 exacerbated the reduction of GSK3β ubiquitination in hTau‐expressing cells (Figure 4G,H). Moreover, a cycloheximide (CHX) chase assay demonstrated that HDAC7 overexpression further impedes the degradation of GSK3β in the presence of hTau (Figure 4I,J). Collectively, these findings suggest that HDAC7 stabilizes GSK3β by facilitating tau‐mediated GSK3β acetylation.

### 2.5 HDAC7 Activates GSK3β by Scaffolding the Tau‐GSK3β Complex Independent of Its Deacetylase Activity

HDAC7 can deacetylate non‐nuclear substrates or act as scaffolds for protein complex via non‐enzymatic function (Kodikara and Pflum 2025; Wang et al. 2024). To explore the functional role of HDAC7 in driving tau‐mediated GSK3β acetylation, we created a putative enzyme‐dead variant of HDAC7 by substituting histidine (H672) with alanine (H672A), a conserved residue required for HDAC7 enzymatic activity (Das Gupta et al. 2020). Co‐IP assays were performed in HEK293T cells expressing GSK3β‐3xFlag, hTau‐GFP and WT or H672A mutated HDAC7 with antibodies against the exogenous GSK3β (Flag) or hTau (Tau13). Analysis of Flag (GSK3β)‐IP and Tau13 (hTau)‐IP samples showed that the interaction between HDAC7 and GSK3β was not affected by the enzyme‐dead mutant, and the interaction between HDAC7 and hTau was also held regardless of HDAC7 enzyme activity (Figure 5A,B). Importantly, the WT and enzyme‐dead mutant HDAC7 equally increased the amount of GSK3β‐binding hTau, as well as the amount of hTau‐binding GSK3β (Figure 5A,C), suggesting that HDAC7 may act as a scaffolding protein for tau‐GSK3β complex formation independent of its deacetylase activity. Next, we assessed whether HDAC7 enzymatic activity affects tau‐mediated GSK3β acetylation. Analysis of Tau13‐IP samples revealed that, in comparison to the vector group, both WT and enzyme‐dead mutant HDAC7 increased the acetylation level of GSK3β interacting with hTau at K15 residue (Figure 5D,E). An equivalent increase of phosphorylated GSK3β at Tyr216 (active) in the Tau13 (hTau)‐IP samples was also observed in both WT and enzyme‐dead mutant HDAC7‐expressing group (Figure 5D,F). Collectively, these data indicate that HDAC7 activates GSK3β via non‐enzymatically scaffolding tau‐GSK3β complex.

**FIGURE 5:**
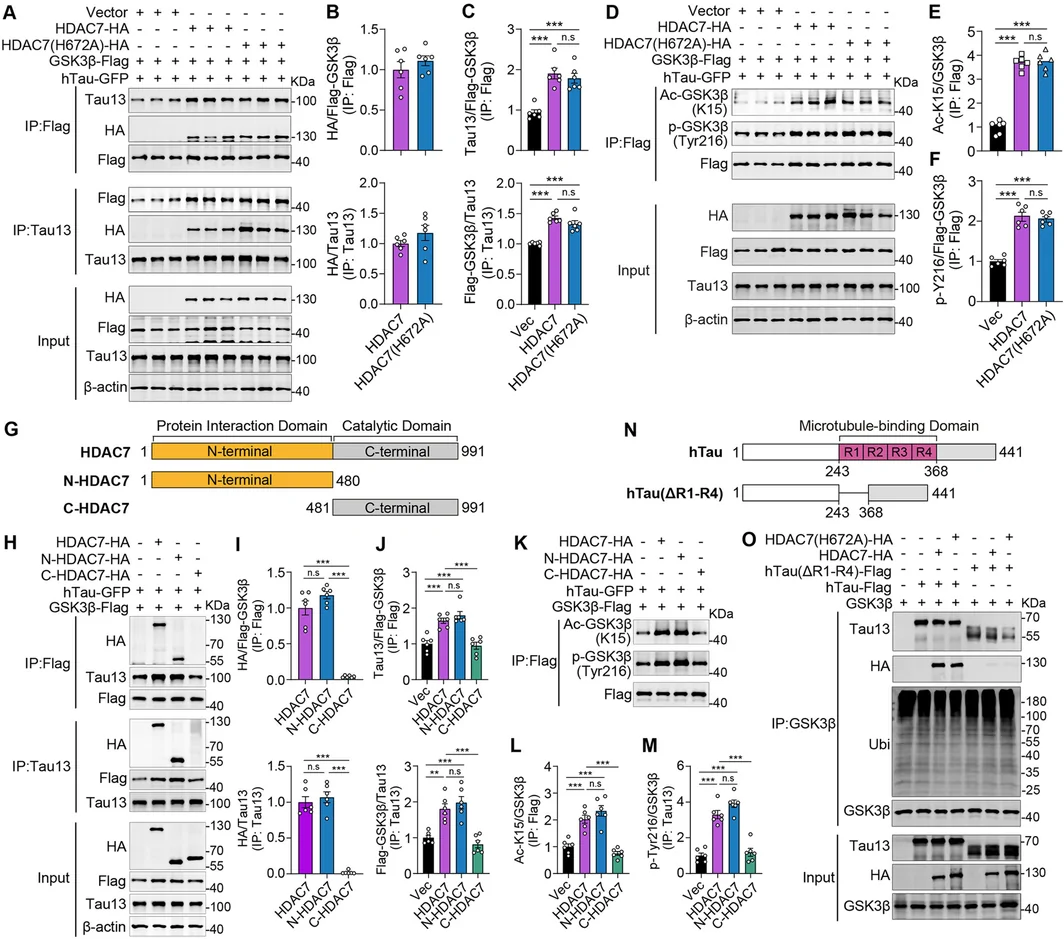
HDAC7 scaffolds GSK3β‐tau interaction to enable tau‐mediated K15 acetylation and GSK3β activation independently of its deacetylase activity. (A) Co‐IP assay analysis of the interaction among HDAC7, GSK3β, and hTau in HEK293T cells co‐transfected with GSK3β‐Flag, hTau‐GFP, and HDAC7‐HA, catalytically inactive HDAC7 (H672A)‐HA, or control vector. GSK3β or hTau was immunoprecipitated with anti‐Flag or anti‐Tau13 antibody, respectively. (B, C) Quantification of the Co‐IP assays: (B) levels of HDAC7 co‐precipitated with GSK3β or tau, (C) levels of tau co‐precipitated with GSK3β and vice versa. *n* = 6 per group. (D) Co‐IP analysis with anti‐Flag antibody showing p‐GSK3β (Tyr216) and ac‐GSK3β (K15) levels in the GSK3β immunoprecipitates. (E, F) Quantification of ac‐GSK3β (K15)/GSK3β and p‐GSK3β (Tyr216)/GSK3β in the IP fractions. *n* = 6 per group. (G) Schematic diagrams of HDAC7 deletion constructs (full‐length, N‐HDAC7: N‐terminal domain of HDAC7; C‐HDAC7: C‐terminal domain of HDAC7). (H) Co‐IP assay analysis of the interaction among HDAC7 truncation mutants, GSK3β, and hTau in HEK293T cells co‐transfected with GSK3β‐Flag, hTau‐GFP, and HDAC7‐HA or N‐HDAC7‐HA or C‐HDAC7 or control vector. GSK3β or hTau was immunoprecipitated with anti‐Flag or anti‐Tau13 antibody, respectively. (I, J) Quantification of the Co‐IP assays: (I) levels of HA‐tagged HDAC7 truncation mutants co‐precipitated with GSK3β or tau, (J) levels of tau co‐precipitated with GSK3β and vice versa. *n* = 6 per group. (K) Co‐IP analysis with anti‐Flag antibody showing the levels of p‐GSK3β (Tyr216) and ac‐GSK3β (K15) in the GSK3β immunoprecipitates. (L, M) Quantification of ac‐GSK3β (K15)/GSK3β and p‐GSK3β (Tyr216)/GSK3β in the IP fractions as shown in J. *n* = 6 per group. (N) Schematic diagrams of hTau deletion constructs (full‐length and ΔR1‐R4). (O) Co‐IP analysis of GSK3β interaction with HDAC7 or hTau, and GSK3β ubiquitination, in HEK293T cells co‐transfected with GSK3β, hTau‐Flag or a tau truncation lacking microtubule‐binding repeat (ΔR1‐R4)‐Flag, and HDAC7‐HA or HDAC7(H672A)‐HA. Statistical significance was determined by one‐way ANOVA with Tukey's post hoc analysis. Data are shown as mean ± SEM, *p < 0.05, **p < 0.01, ***p < 0.001, n.s., not significant.

Additionally, we explored the effect of HDAC7 enzymatic activity on GSK3β activity in vivo. WT mice expressing hTau were administered intraperitoneally with TMP195, a selective class IIa HDAC inhibitor known for its ability to inhibit HDAC7 activity (Ye et al. 2025). Western blotting analysis revealed that although the levels of p‐GSK3β (Tyr216) were elevated in hTau group mice compared to control GFP group mice, this activation was unaffected by TMP195 treatment (Figure S6). The in vivo data further corroborate that tau‐mediated GSK3β activation is independent of HDAC7 enzymatic activity.

To further elucidate the contribution of the HDAC7‐GSK3β‐tau complex to tau pathological phosphorylation, we measured the levels of pathology‐associated phosphorylated tau regulated by GSK3β. In cells overexpressing hTau, both the WT and the enzyme‐dead mutant HDAC7 (H672A) resulted in elevated levels of phosphorylated tau at sites p‐Ser202, p‐Thr205, p‐Ser214, p‐Thr217, p‐Thr231, and p‐Ser396, with comparable efficiency (Figure S7A,B). This observation suggests that the increased GSK3β‐associated tau phosphorylation by HDAC7 is independent of its deacetylase activity.

To further clarify the specific domain of HDAC7 responsible for its scaffolding function, we generated N‐terminal (N‐HDAC7, aa 1–480) and C‐terminal (C‐HDAC7, aa 481–991) truncation mutants of HDAC7 (Figure 5G). Co‐IP assays revealed that N‐HDAC7, which retains the N‐terminal interaction domain but lacks the C‐terminal catalytic domain, retained full scaffolding function comparable to full‐length HDAC7, including binding to both GSK3β and tau (Figure 5H,I), facilitating GSK3β‐tau complex formation (Figure 5H,J), and enhancing GSK3β K15 acetylation and Tyr216 phosphorylation (Figure 5K–M). In contrast, C‐HDAC7, which comprises solely the catalytic domain, was unable to bind GSK3β, failed to mediate the interaction between tau and GSK3β, and did not enhance GSK3β activation (Figure 5G–M). Consequently, N‐HDAC7, but not C‐HDAC7, significantly increased tau phosphorylation at p‐Ser202, p‐Thr205, p‐Ser214, p‐Thr217, p‐Thr231, and p‐Ser396 (Figure S7C,D). These results indicate that the N‐terminal domain of HDAC7 is critical for scaffolding the GSK3β‐tau interaction and facilitating downstream signaling, independent of the C‐terminal catalytic domain.

Previous studies suggest that the microtubule‐binding domain (MBD) of tau is crucial for its acetyltransferase activity and its interaction with GSK3β (Cohen et al. 2013; Zhou et al. 2022). To determine whether this tau domain influences the formation of the HDAC7‐GSK3β‐tau complex, we created a variant of hTau by deleting the MBD region (ΔR1‐R4) (Figure 5N). Analysis of Flag (GSK3β)‐IP samples revealed that the deletion of the tau MBD region diminished the interactions between HDAC7 and GSK3β, as well as between tau and GSK3β (Figure 5O). Furthermore, deletion of the MBD region restored the reduction in GSK3β ubiquitination level induced by HDAC7 (Figure 5O). These findings underscore the pivotal role of the tau MBD region in the assembly of the HDAC7‐GSK3β‐tau complex and the consequent stability of GSK3β.

### 2.6 HDAC7 Deletion Attenuates GSK3β Activation and Stability and Reducing Tau Phosphorylation in Mouse Brain

To evaluate whether reduction of HDAC7 can suppress GSK3β activation and consequently delay tau phosphorylation progression, we employed an AAV‐mediated Cre‐loxP system to genetically ablate neuronal Hdac7 in mouse brain. Specifically, AAV‐hSyn‐hTau‐GFP and AAV‐hSyn‐Cre‐NLS‐mCherry or control AAV‐hSyn‐mCherry were co‐infused into the hippocampal CA3 region of *Hdac7* ^*flx*/*flx*^ mice (Figure 6A). Immunofluorescence staining confirmed the expression of hTau and the successful deletion of HDAC7 2 months post‐AAV injection (Figure 6B). Immunoprecipitation with anti‐Tau13 antibody showed that hTau co‐precipitated with endogenous GSK3β in *Hdac7* ^+/+^ mice, whereas this interaction was markedly diminished in *Hdac7* ^−/−^ mice (Figure S8), confirming that HDAC7 is essential for the GSK3β‐tau interaction in the brain. We then examined the consequences of HDAC7 deletion on GSK3β acetylation and activation. The acetylation levels of GSK3β at K15 were increased in hTau‐expressing mice as compared to vector‐injected controls, corroborating our in vitro findings. Notably, deletion of HDAC7 resulted in a significant reduction in acetylated GSK3β (K15) levels in hTau‐expressing mice (Figure 6C,D). GSK3β activation in hTau‐expressing mice was significantly attenuated following HDAC7 deletion, evidenced by an increase in p‐GSK3β (Ser9) and a decrease in p‐GSK3β (Tyr216) (Figure 6C,E,F). Total GSK3β protein level was also reduced by HDAC7 deletion in hTau‐expressing mice (Figure 6C,G), with its mRNA level remaining consistent across all experimental groups (Figure 6H). Furthermore, immunostaining analysis demonstrated that HDAC7 knockout substantially attenuated the expression levels of total GSK3β and activity‐related p‐GSK3β (Tyr216) in hTau‐expressing neurons within the hippocampal CA3 region (Figure 6I,J). These data suggest that HDAC7 deletion exerts an inhibitory effect on both GSK3β kinase activity and protein stability in the context of tau pathology.

**FIGURE 6:**
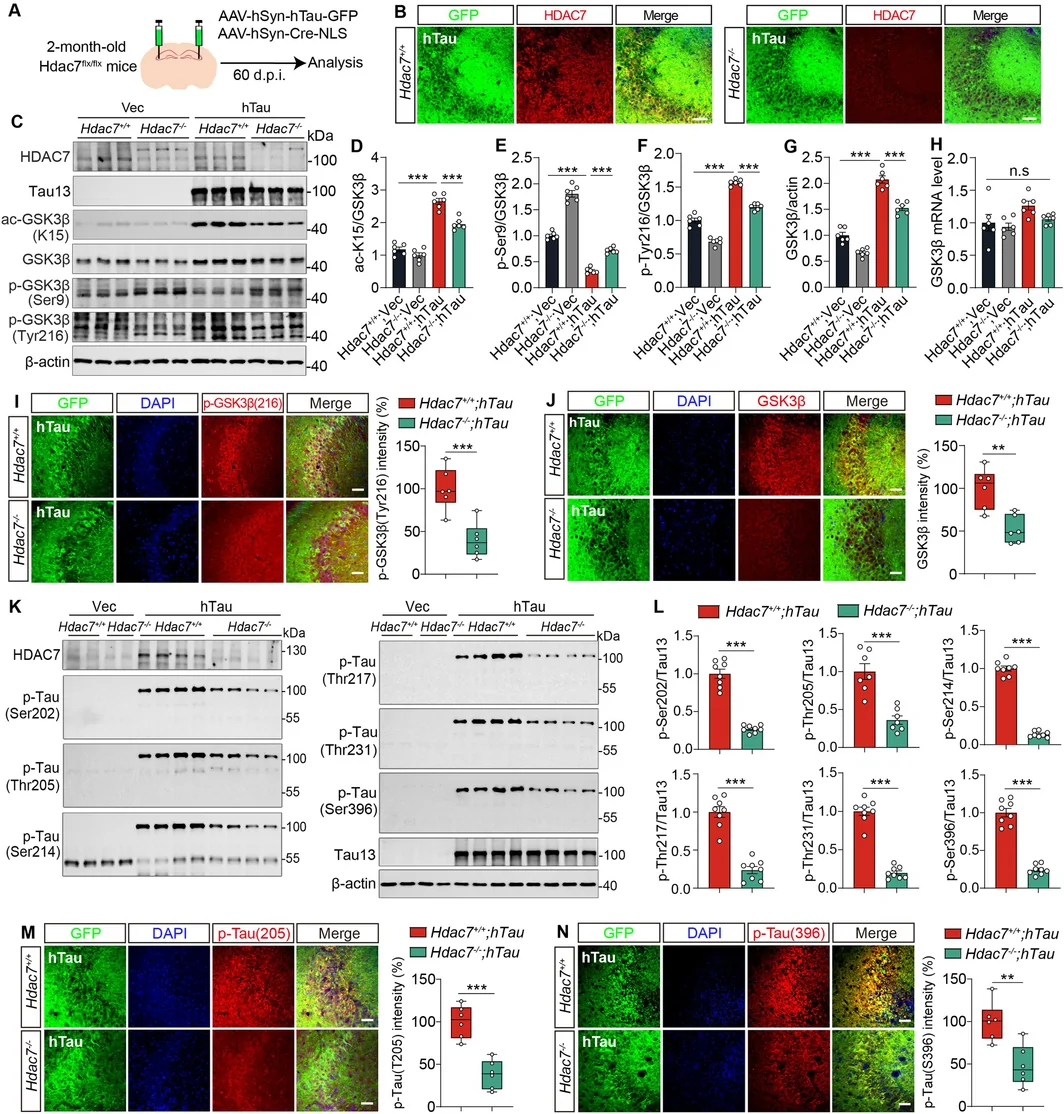
Neuronal HDAC7 deletion attenuates GSK3β activation and tau hyperphosphorylation in hTau‐expressing mice. (A) Experimental timeline for AAV infusion in *Hdac7* ^*flx*/*flx*^ mice. (B) Representative immunofluorescence image showing hTau‐GFP expression and HDAC7 deletion in the hippocampal CA3 region at 2 months. Scale bars: 80 μm. (C) Western blot analysis of HDAC7, Tau13, ac‐GSK3β (K15), p‐GSK3β (Ser9), p‐GSK3β (Tyr216), and total GSK3β in the hippocampal CA3 extracts from *Hdac7* ^*flx*/*flx*^ mice with neuronal hTau expression and HDAC7 deletion. (D–G) Quantification of the blots in (C): (D) ac‐GSK3β (K15)/GSK3β, (E) p‐GSK3β (Ser9)/GSK3β, (F) p‐GSK3β (Tyr216)/GSK3β, (G) GSK3β/actin. *n* = 6 mice per group. (H) RT‐qPCR analysis of GSK3β mRNA levels in the hippocampal CA3 tissues. *n* = 6 mice per group. (I, J) Representative immunofluorescence staining images and quantification of (I) p‐GSK3β (Tyr216) and (J) total GSK3β in the hippocampal CA3 region of hTau‐expressing mice. *n* = 6 mice per group. Scale bar: 40 μm. (K, L) Western blot analysis and quantification of phosphorylated tau (p‐Ser202, p‐Thr205, p‐Ser214, p‐Thr217, p‐Thr231, p‐Ser396) and total human tau (Tau13) in the hippocampal CA3 extracts. *n* = 6 mice per group. (M, N) Representative immunofluorescence staining images and quantification of (M) p‐Tau (Thr205) and (N) p‐Tau (Ser396) in the hippocampal CA3 region of hTau‐expressing mice. *n* = 6 mice per group. Scale bar: 40 μm. Statistical significance was determined by unpaired Student's *t*‐test (I–N) or one‐way ANOVA with Tukey's post hoc analysis (D–H). Data are shown as mean ± SEM, *p < 0.05, **p < 0.01, ***p < 0.001, n.s., not significant.

Consistent with these findings, western blot analysis showed that the phosphorylation levels of hTau at GSK3β‐associated sites, including p‐Ser202, p‐Thr205, p‐Ser214, p‐Thr217, p‐Thr231, and p‐Ser396, were significantly reduced in Hdac7 knockout mice (Figure 6K,L). Immunostaining of p‐Tau (Thr205) and p‐Tau (Ser396) further confirmed that HDAC7 deletion decreased tau phosphorylation in the hippocampus (Figure 6M,N). Collectively, these data support our conclusion that the deletion of HDAC7 attenuates GSK3β kinase activity and protein stability, thereby inhibiting AD‐like tau phosphorylation.

### 2.7 HDAC7 Deletion Ameliorates hTau‐Induced Synaptic Impairments and Cognitive Decline in Mice

Tau hyperphosphorylation is highly associated with synaptic loss and behavioral abnormalities (Watamura et al. 2025; Ye, Yin, Yin, et al. 2020). By electron microscopy analysis, we observed that HDAC7 deletion led to a significant increase in the number of synapses within the hippocampal CA3 region of hTau‐expressing mice (Figure 7A,B). This synaptic improvement was further supported by Western blot analysis, which demonstrated that HDAC7 deletion restored the levels of presynaptic proteins, such as synaptophysin (Syp) and synapsin‐1 (Syn‐1), as well as postsynaptic proteins, including PSD95 and the AMPA receptor subunit GluA1, in hTau‐expressing mice (Figure 7C,D). Behavioral tests were performed for evaluating cognitive function. The MWM behavioral test indicated that neuronal HDAC7 deletion significantly alleviated spatial learning and memory deficits in hTau‐expressing mice, as evidenced by a reduced latency to reach the platform (Figure 7E–G), increased exploration time in the target zone (Figure 7H), and a higher frequency of crossings over the target platform (Figure 7I), while swimming speed remained comparable across all experimental groups (Figure 7J). Furthermore, hTau‐expressing mice with HDAC7 deletion demonstrated an increased preference for the novel object in the NOR test, indicating improved cognitive function (Figure 7K,L). Together, these data support that HDAC7 deletion can effectively ameliorate synaptic loss and cognitive deficits in hTau‐expressing mouse model.

**FIGURE 7:**
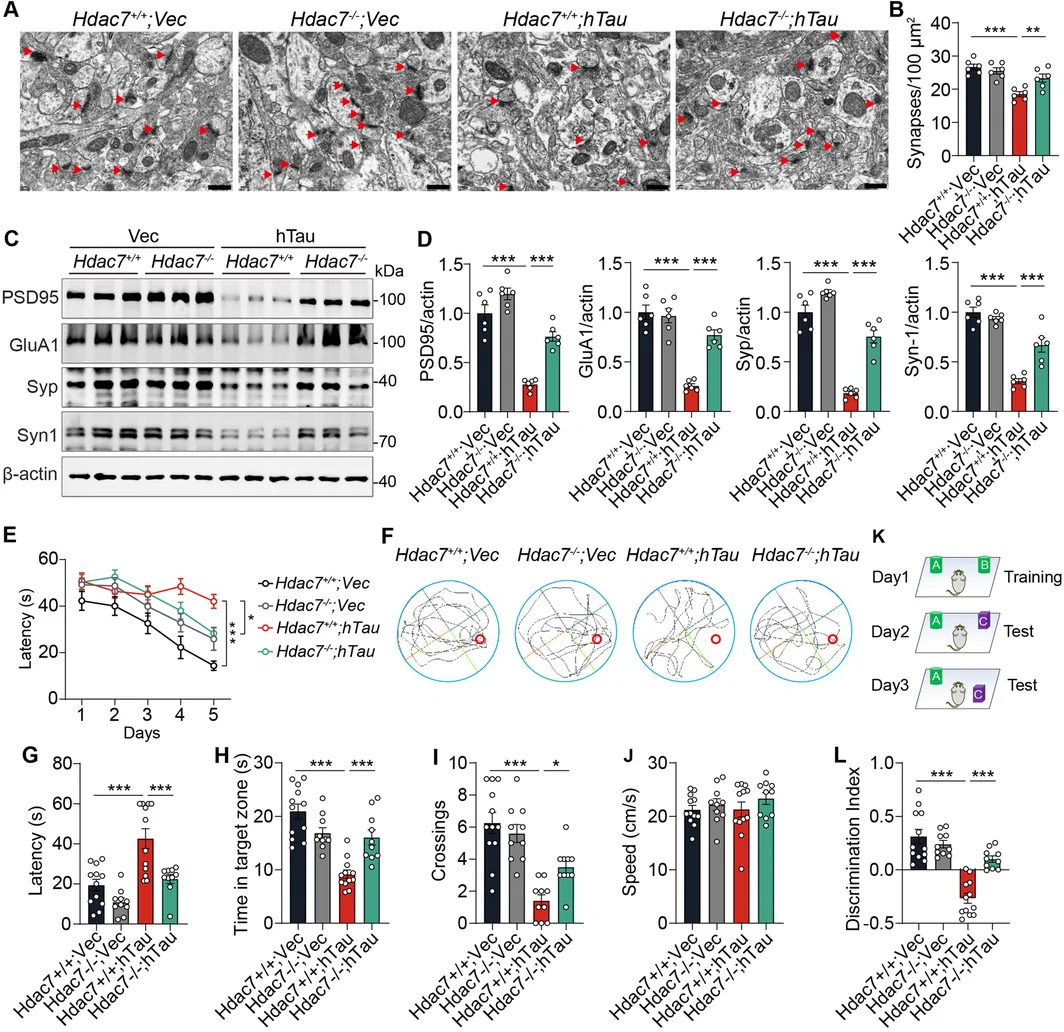
Neuronal HDAC7 deletion ameliorates synaptic impairments and cognitive deficits in hTau‐expressing mice. (A, B) Representative electron microscopy images and quantification of the synapses in the hippocampal CA3 region from *Hdac7* ^*flx*/*flx*^ mice with neuronal hTau expression and HDAC7 deletion. *n* = 6 mice per group, ≥ 10 micrographs per mouse were analyzed. (C, D) Western blot analysis of synaptic proteins PSD95, GluA1, synaptophysin, and synapsin‐1 in the hippocampal CA3 extracts. *n* = 6 mice per group. (E–J) MWM behavioral test. (E) Escape latency during the training phase, (F) representative travel traces of the mice, (G) first latency to reach the platform, (H) time spent in the target zone, (I) platform crossings, and (J) swimming speed during the probe test. (K) Schematic timeline of the novel object recognition (NOR) test. (L) Discrimination index of mice in the probe trial of NOR test. For behavioral tests, *n* = 12 (*Hdac7* ^+/+^; *Vec*), 10 (*Hdac7* ^−/−^; *Vec*), 12 (*Hdac7* ^+/+^; *hTau*), 10 (*Hdac7* ^−/−^; *hTau*) mice per group. Statistical significance was determined by one‐way ANOVA with Tukey's post hoc analysis. Data are shown as mean ± SEM, *p < 0.05, **p < 0.01, ***p < 0.001.

## 3 Discussion

Mounting evidence suggests that increased activity of GSK3β constitutes a crucial step in the pathological phosphorylation of tau, thereby initiating downstream NFT formation and neurodegeneration (Chakraborty et al. 2024; Gong et al. 2011). Despite the generally unstable and transient nature of enzyme‐substrate interactions, and the fact that tau is not the sole substrate for GSK3β, the frequently observed colocalization/interaction between GSK3β and tau during AD pathogenesis is thought provoking. In this study, our results suggest that the formation of the HDAC7‐GSK3β‐tau complex plays a pivotal role in stabilizing and activating tau‐binding GSK3β in an Alzheimer‐like tau mouse model. Our findings indicate that HDAC7 functions as a scaffolding protein, stabilizing the interaction between GSK3β and tau, and facilitating tau‐mediated GSK3β acetylation, which leads to GSK3β activation and protein stability. This process occurs independently of HDAC7's deacetylase activity. Overexpression of HDAC7 enhances GSK3β stability and activation, as well as tau phosphorylation in hTau‐expressing mice, whereas HDAC7 deletion reduces GSK3β protein levels and kinase activity, mitigates tau phosphorylation, and restores synaptic and cognitive functions in hTau‐expressing mice. Our findings underscore the importance of the HDAC7‐GSK3β‐tau complex in promoting disease‐related tau phosphorylation and cognitive decline, suggesting the potential of HDAC7 reduction as a therapeutic strategy for AD and other tauopathies.

Dysregulation of HDACs is correlated with disease phenotypes in a wide range of AD and tauopathy models (Rodrigues et al. 2020; Trzeciakiewicz et al. 2020). To better understand which HDACs are most responsible for hTau‐associated neuropathology, we screened HDAC mRNA levels in hTau‐expressing mouse models. In this study we found that HDAC7, a class IIa HDAC family member, is significantly upregulated in the cytosol of hTau‐expressing neurons. Through neuron‐specific overexpression of HDAC7, we observed an exacerbated tau phosphorylation and behavioral deficits in hTau‐expressing mice. Using a comprehensive screening of kinases and phosphatases associated with tau phosphorylation both in vivo and in vitro, we attributed the enhanced tau phosphorylation to the increased activity and stability of GSK3β. This was evidenced by an elevated p‐GSK3β (Tyr216)/GSK3β ratio and an increased total GSK3β protein level, despite unchanged mRNA expression. Although increased CDK5 activity and decreased PP2Ac levels were also observed in HDAC7‐overexpressing mice, these changes were not replicated in cultured neurons with HDAC7 overexpression. Furthermore, the AD‐associated tau phosphorylation sites p‐Thr217 and p‐Ser262, which are exclusively catalyzed by GSK3β and not by CDK5 (Hanger et al. 2009), were significantly elevated by HDAC7 overexpression in the hippocampus, further pointing to the contribution of GSK3β activation in accelerating tau phosphorylation following HDAC7 overexpression. Importantly, HDAC7 is also upregulated in the brains of AD patients. Our immunofluorescence analysis demonstrated elevated HDAC7 levels co‐localized with AT8‐positive tau aggregates, corroborating previous studies that reported increased HDAC7 mRNA levels in AD brains (Ye et al. 2025), and identified it as a hub gene in AD co‐expression networks (Cao et al. 2024). Colocalization analysis showed that the extent of HDAC7‐AT8 overlap was greater in hTau mice compared to AD brains, likely due to the uniform, overexpression‐driven pathology in the mouse model compared to the chronic, heterogeneous, and multi‐regional characteristics of human AD. These findings align with the selective upregulation of HDAC7 we observed in hTau‐expressing neurons.

Given the progressive escalation of GSK3β activation and tau pathological phosphorylation in the brain of AD patients, elucidating the molecular mechanisms that regulate the GSK3β‐tau interaction is crucial for understanding tau pathogenesis. Previous studies have indicated that GSK3β is bound to tau within a microtubule‐associated complex (Sun et al. 2002), where 14–3‐3 facilitates GSK3β‐mediated tau phosphorylation (Yuan et al. 2004). Our study identifies HDAC7 as a direct binding partner of both tau and GSK3β, forming an HDAC7‐GSK3β‐tau complex that facilitates the structural proximity necessary for their functional interaction. Notably, tau protein exhibits acetyltransferase activity and is capable of catalyzing the acetylation of GSK3β at K15 residues, resulting in the upregulation and activation of the kinase (Zhou et al. 2022). Building on these studies, we further investigated the role of HDAC7 in this biochemical process. Our data indicate that HDAC7 functions as a scaffolding protein, linking tau to GSK3β, enhancing tau‐mediated acetylation of GSK3β at K15. This acetylation event not only stabilizes the kinase by inhibiting its ubiquitination but also promotes a phosphorylation switch, increasing Tyr216 phosphorylation (active form) while decreasing Ser9 phosphorylation (inactive form), thereby enhancing its intrinsic catalytic activity (Zhou et al. 2022). Moreover, we showed that deletion of the tau acetyltransferase activity domain (MBD region) restores the ubiquitination status of GSK3β in the presence of HDAC7, further supporting the necessity of tau's catalytic activity for HDAC7‐mediated GSK3β activation. These findings hold significance in degenerated neurons in AD patients, where the active form of GSK3β colocalizes with tau when an MBD region‐targeting antibody is used (Leroy et al. 2002). GSK3β directly phosphorylates tau protein at disease‐associated epitopes, thereby contributing to tau pathological aggregation (Chakraborty et al. 2024). Our data revealed that the overexpression of HDAC7 exacerbates tau phosphorylation at multiple GSK3β‐associated sites, including p‐Ser205, p‐Ser214, and p‐Ser396, which are also abnormally hyperphosphorylated in the brain of AD patients (Ye et al. 2024). Previous studies have indicated that the phosphorylation status of the tau MBD region may initiate a conformational change in tau, thereby enhancing its acetyltransferase activity, which facilitates the acetylation of both GSK3β and tau itself (Cohen et al. 2013; Zhou et al. 2022). Considering the increases in tau Ser262 phosphorylation, tau K174 acetylation, and GSK3β K15 acetylation following HDAC7 overexpression in both in vivo and in vitro models, we propose a bidirectional regulatory crosstalk between GSK3β acetylation and tau phosphorylation/acetylation. In this proposed model, HDAC7‐scaffolded the hyperactivation of GSK3β, facilitated by HDAC7, drives tau hyperphosphorylation, which subsequently enhances tau's acetyltransferase activity towards both GSK3β and tau itself, leading to increased GSK3β K15 acetylation and tau self‐acetylation. This self‐amplifying cycle perpetuates GSK3β activation and pathological tau modifications during AD pathogenesis, with HDAC7 serving as a scaffold factor that sustains this process.

Our findings indicate that HDAC7 catalytic activity is dispensable for scaffolding the GSK3β‐tau interaction. Both the catalytically inactive H672A mutant and the catalytic domain deletion mutant were able to facilitate the formation of the GSK3β‐tau complex, GSK3β K15 acetylation, and subsequent tau phosphorylation with efficacy comparable to that of WT HDAC7. In contrast, the N‐terminal deletion mutant eliminated GSK3β binding and tau bridging. Furthermore, our findings indicated that pharmacological inhibition of HDAC7 with TMP195 did not attenuate GSK3β activation in hTau‐expressing neurons. Collectively, these data demonstrate that HDAC7 functions through its N‐terminal protein interaction domain, independent of its C‐terminal catalytic activity. Class IIa HDACs, including HDAC7, exhibit context‐dependent functions. They can deacetylate specific non‐nuclear substrates in certain cell types. For example, HDAC7 deacetylates PINK1 and TFEB in astrocytes (Ye et al. 2025; Yue et al. 2025), while HDAC9 targets IKKα/β in macrophages and endothelial cells (Asare et al. 2020). Conversely, these enzymes also act as pseudodeacetylases, influencing cellular processes through scaffolding mechanisms that are independent of their catalytic activity (Kodikara and Pflum 2025). This is illustrated by HDAC5, which recruits HDAC1/Sin3a co‐repressor complexes to cardiac gene promoters (Harris et al. 2016), and HDAC4, which links transcription factors with HDAC3 (Lee et al. 2015). Moreover, HDAC7 has been shown to activate 6‐phosphogluconate dehydrogenase via its N‐terminal protein–protein interaction domain, independent of its catalytic activity (Wang et al. 2024). In line with this paradigm, overexpression of HDAC7 did not directly deacetylate GSK3β or tau; rather, it facilitated tau‐mediated GSK3β acetylation through its scaffolding function, further corroborating the non‐enzymatic mechanism proposed here.

The HDAC7‐GSK3β‐tau scaffolding mechanism proposed in this study is primarily based on overexpression systems, a common limitation when dissecting protein complex formation. Although our data suggest that HDAC7 is required for the GSK3β‐tau interaction in vivo, this methodology still depends on the AAV‐based human tau overexpression model. The AAV‐hTau model recapitulates key features of human tauopathy (Ye, Yin, Yin, et al. 2020; Yin et al. 2016), supporting its utility for mechanistic investigation. Nonetheless, its acute, focal delivery does not fully mimic the progressive, multi‐regional tau pathology characteristic of human tauopathy, nor does it account for the contributions of other tau isoforms present in human disease (Szegvari et al. 2025). Whether this scaffolding mechanism operates on endogenous GSK3β‐tau complexes in the brain of AD patients or other human tauopathies warrants further investigation.

The improvement of synaptic and cognitive functions in hTau‐expressing mice following HDAC7 deletion was associated with a decrease in the levels of phosphorylated tau. The attenuated GSK3β activity further demonstrated the functional implications of HDAC7 deletion in the brain. While our data identify GSK3β as the primary mediator of HDAC7‐driven tau pathology, the potential involvement of other pathways in the protective effects of HDAC7 deletion cannot be excluded. For example, HDAC7 deletion or knockdown has also been shown to attenuate NF‐κB‐associated inflammatory responses and enhance TFEB‐associated lysosomal degradation in the brain (Ye et al. 2022), suggesting that these mechanisms may partially contribute to the neuroprotective effects of HDAC7 deletion. Notably, overexpression of HDAC7 alone resulted in cognitive deficits without overt changes in endogenous murine tau phosphorylation, yet it increased GSK3β activity. This suggests that HDAC7‐driven GSK3β activation may impair cognition through tau‐independent mechanisms. GSK3β can phosphorylate PSD95 and regulate AMPA receptor trafficking (C. D. Nelson et al. 2013; Peineau et al. 2007), thereby modulating synaptic plasticity, which might contribute to HDAC7‐induced cognitive impairments. Furthermore, HDAC7 disrupts mitochondrial function through promoting mitochondrial fission and impairing mitophagy (Abrol et al. 2025; Yue et al. 2025), processes that may also play a role in HDAC7‐mediated cognitive dysfunction. It is important to note that the synaptic improvements were inferred from morphological and biochemical analyses rather than direct electrophysiological measurements. Our study indicates that HDAC7 bridges GSK3β and tau, acting directly on the GSK3β‐tau phosphorylation complex. Although GSK3β plays essential physiological roles in metabolism and immune function (Kim et al. 2022; H. Zhou et al. 2018) and its direct pharmacological inhibition has shown limited clinical benefit (Lovestone et al. 2015), targeting the HDAC7 scaffold rather than GSK3β catalytic activity may offer a more selective therapeutic approach. While HDAC7 reduction in this study was achieved through genetic manipulation, recent investigations have focused on developing selective HDAC7 degraders to address its non‐enzymatic functions. Consequently, the discovery of a brain‐permeable small molecule HDAC7 degrader could represent a promising therapeutic strategy for AD treatment by targeting the GSK3β‐tau pathological cycle.

## 4 Methods and Materials

### 4.1 Viruses, Reagents and Antibodies

The plasmids pEGFP‐hTau (human 2N4R MAPT), pEGFP‐C1, pcDNA3.1‐hTau (human wild‐type 2N4R MAPT)‐3xFlag, pcDNA3.1‐MAPT (ΔR1‐R4)‐3xFlag, pcDNA3.1–3xFlag, pEGFP‐HDAC7, pcDNA3.1‐HDAC7‐HA, pcDNA3.1‐HDAC7 (H672A)‐HA, pcDNA3.1‐N‐HDAC7‐HA, pcDNA3.1‐C‐HDAC7‐HA, pcDNA3.1‐GSK3β‐3xFlag, pcDNA3.1‐GSK3β (K15R)‐3xFlag, and pcDNA3.1‐GSK3β were synthesized by Obio Technology (Shanghai, China). The adeno‐associated virus (AAV) constructs, including AAV‐hSyn‐hTau‐GFP, AAV‐hSyn‐GFP, AAV‐hSyn‐HDAC7‐3xFlag, AAV‐hSyn‐3xFlag, AAV‐hSyn‐Cre‐NLS‐mCherry, and AAV‐hSyn‐mCherry, were developed by Obio Technology (Shanghai, China). The AAV2/8 serotype was used in animal experiments. TMP195 (a selective class IIa HDAC inhibitor, S8502) and Cycloheximide (CHX, S7418) were procured from Selleck (Houston, TX, USA). The antibodies used in this study are listed in supplementary Table S1.

### 4.2 Animals, Stereotaxic Surgery and Drug Treatment

*Hdac7* ^*flx*/*flx*^ mice (C57BL/6JGpt‐Hdac7^em1Cflox/Gpt^, Strain NO. T069577) were procured from GemPharmatech Co. Ltd. Human tau transgenic mice (B6.Cg‐Tg (MAPT) 8cPdav *Mapt* ^*tm1(EGFP)klt*^/J, Strain NO. 005491) were acquired from the Jackson Laboratory. Male C57BL/6 mice were sourced from Changzhou Cavens Laboratory Animal Co. Ltd. (Changzhou, China). The mice were housed in groups of four to five per cage, with unrestricted access to food and water, and were maintained under a 12‐h light/dark cycle (lights on at 7:00 p.m. and off at 7:00 a.m.) at a constant temperature of 22°C ± 2°C. This study adhered to all relevant ethical regulations concerning animal testing and research. All procedures were approved by institutional guidelines and the Animal Care and Use Committee (Shenzhen University, Shenzhen, China) of the university's animal core facility.

For brain stereotactic injection, mice were anesthetized with isoflurane and subsequently positioned on a stereotaxic apparatus. The surgical area was sterilized with iodophor, and an incision was made along the midline of the scalp. Two holes were drilled bilaterally at posterior 2.1 mm, lateral ±2.5 mm relative to the bregma. A volume of 0.5 μL of either AAV‐hSyn‐hTau‐GFP (5.1 × 10^12^ vg/mL) or AAV‐hSyn‐GFP (4.8 × 10^12^ vg/mL), AAV‐hSyn‐HDAC7‐3xFlag (4.0 × 10^12^ vg/mL) or AAV‐hSyn‐3xFlag (6.1 × 10^12^ vg/mL), AAV‐hSyn‐Cre‐NLS‐mCherry (4.5 × 10^12^ vg/mL) or AAV‐hSyn‐mCherry (5.5 × 10^12^ vg/mL) were injected into each site of the dorsal CA3 (ventral −2.2 from the skull) using an automatic microinjection system (World Precision Instruments, USA) at a controlled rate of 50 nL/min. The injection needle was retained in position for 5 min post‐injection before withdrawal. The incision was then sutured, and the mice were placed adjacent to a heating source to facilitate recovery.

For intraperitoneal administration, TMP195 was diluted at a concentration of 7.5 mg/mL in sterile 0.9% saline, with the addition of 5% (v/v) Tween‐80 and 20% (v/v) PEG‐300. Four weeks post‐injection with AAV‐hSyn‐hTau‐GFP or AAV‐hSyn‐GFP, mice received intraperitoneal injections of the diluted TMP195 at a dosage of 50 mg/kg every 3 days for a duration of 1 month.

### 4.3 Morris Water Maze (MWM) Test

The MWM test was performed as previously described (Ye et al. 2025). Mice were trained for five consecutive days (three trials/day, 60 s max per trial). Spatial memory was assessed 48 h after the last training session by removing the platform and recording latency to first platform crossing, platform crossings, and time in target quadrant during a 60‐s probe trial.

### 4.4 Novel Object Recognition (NOR) Test

The NOR test was conducted as previously described (Ye et al. 2025). Mice were habituated to the arena for 3 min/day over three consecutive days, followed by a 5‐min training session with two identical objects. After 24 h, one object was replaced with a novel object, and exploration time was recorded for 5 min. The discrimination index was calculated as (C − A)/(C + A), where C and A represent exploration time for the novel and familiar objects, respectively.

### 4.5 Cell Culture and Treatment

Primary cortical neurons were isolated from WT C57BL/6 mice embryos (E17‐E19) as described previously (Ye, Yin, Liu, et al. 2020). Cortical tissues were dissected and gently minced in Hank's buffered saline solution, followed by digestion with 0.125% trypsin at 37°C for 15 min. The dissociated cells were seeded in F‐12 medium supplemented with 10% fetal bovine serum (FBS) on poly‐D‐lysine‐coated coverslips. Four hours post‐seeding, the medium was replaced with Neurobasal medium supplemented with 2% B27 and 1% GlutaMAX. On day in vitro 5, neurons were infected with AAV at a MOI of 10,000. Plasmid transfection in DIV7 primary neurons was performed using LipoFactMax (ABP Biosciences, Beltsville, USA), and the medium was replaced with fresh maintenance medium 6 h post‐transfection.

HEK293T cells were cultured with DMEM‐high glucose medium supplemented with 10% Fetal FBS and 1% penicillin/streptomycin at 37°C in the presence of 5% CO_2_. HEK293T cell transfection was performed with lipoFactMax.

### 4.6 Western Blotting

The cells were harvested and lysed with RIPA buffer (Beyotime, cat# P0013B) on ice for 10 min, followed by centrifugation at 12,000 *g* for 10 min at 4°C. The hippocampal CA3 region was dissected from mouse brains using a vibrating microtome (Leica, VT1000S, Germany) in ice‐cold PBS following the mouse brain atlas and previously established methods. The CA3 tissue was homogenized with RIPA lysis buffer (Beyotime, cat# P0013B) supplemented with 1% protease inhibitors and phosphatase inhibitors, and subsequently centrifuged at 12,000 *g* for 15 min at 4°C. The supernatant was then transferred to new tubes. Protein concentration was determined using the BCA method (Pierce BCA protein assay kit, 23,225) and samples were denatured in SDS loading buffer. Following SDS‐PAGE, proteins were transferred onto nitrocellulose membranes (Whatman) and blocked with blocking buffer (Beyotime, cat# P0023B) for 1 h at room temperature. Primary antibodies were incubated overnight at 4°C, and corresponding peroxidase‐conjugated secondary antibodies were applied for 1 h at room temperature. Protein signals were detected using the ECL detection system and analyzed with ImageJ software. The antibodies utilized for Western blotting are detailed in supplementary Table S1.

### 4.7 Dot Blotting

Acetylated GSK3β K15 peptide and the corresponding non‐acetylated peptide were synthesized and spotted onto nitrocellulose membranes. The membranes were subsequently air‐dried and blocked with 5% BSA in Tris‐buffered saline with Tween 20 for 1 h at room temperature. Following blocking, the membranes were incubated with the Ac‐GSK3β (K15) antibody at 4°C overnight. After thorough washing, membranes were incubated with HRP‐conjugated secondary antibody for 1 h at room temperature, and signals were detected by the ECL detection system.

### 4.8 Co‐Immunoprecipitation (Co‐IP)

Cultured cells were lysed on ice for 30 min using IP buffer (Beyotime, cat# P0013) supplemented with 1% protease and phosphatase inhibitors. The lysates were subsequently centrifuged at 14,000 *g* for 10 min. Protein concentration was determined using the BCA method (Pierce BCA protein assay kit, catalog number 23225). The supernatants, containing 1 μg of protein, were incubated overnight at 4°C with specific primary antibodies under gentle rocking conditions. This was followed by a 2‐h incubation with protein A/G beads at 4°C. The immunoprecipitates were washed three times with PBS, resuspended in 2 × loading buffer, eluted by boiling for 10 min, and analyzed via immunoblotting. The antibodies employed for co‐immunoprecipitation are listed in supplementary Table S1.

### 4.9 Human Brain Samples

Postmortem human brain specimens were supplied by Dr. Chao Ma from the Human Brain Bank at the Chinese Academy of Medical Sciences and Peking Union Medical College, Beijing, China. AD was diagnosed in accordance with the criteria established by the Consortium to Establish a Registry for Alzheimer's Disease (CERAD) and the National Institute on Aging (NIA). Details regarding the human samples are presented in supplementary Table S2.

### 4.10 Immunofluorescence Staining

For animal experiments, mice were subjected to anesthesia using a 1% solution of pentobarbital sodium, followed by intracardial perfusion with saline and subsequently with a 4% solution of paraformaldehyde (PFA) in 0.1 M phosphate buffer at a pH of 7.4. The brains of the mice were then extracted and further fixed in a 4% PFA solution for a duration of 12 h, after which they were cryoprotected using successive solutions of 20% and 30% sucrose. Brain sections with a thickness of 35 μm were obtained using a cryostat microtome (CM1900, Leica, Germany). Brain sections or cultured cells were fixed with 4% PFA, permeabilized with 0.3% Triton X‐100, and blocked with immunostaining blocking buffer (Beyotime). Samples were incubated with primary antibodies overnight at 4°C, followed by Alexa‐Fluor‐conjugated secondary antibodies (1 h, 37°C) and DAPI counterstaining. Images were acquired using a Zeiss LSM880 confocal microscope. Antibody details are listed in supplementary Table S1.

### 4.11 GSK3β Activity Assay

GSK3β kinase activity was measured using a kit (Genmed, Cat# GMS50161.4.3) according to the manufacturer's instructions. Briefly, hippocampal tissue lysates were incubated with reaction buffer containing the GSK3β substrate at 30°C. Absorbance was recorded at 0 and 5 min, and relative GSK3β activity was calculated as described in the kit manual.

### 4.12 Transmission Electron Microscopy

After inducing deep anesthesia, mice were subjected to transcardial perfusion using a solution composed of 2% glutaraldehyde and 3% paraformaldehyde in PBS. Subsequently, hippocampal slices were fixed in 2.5% glutaraldehyde at pH 7.4 for a period of 2 h. The samples were then rinsed three times with 0.1 M phosphate buffer at pH 7.2 and fixed in 1% osmium tetroxide at 4°C for 2 h. Post‐fixation, the samples underwent dehydration through a graded series of ethanol concentrations. Thereafter, the samples were embedded in Epon‐Araldite resin to ensure adequate penetration and placed in molds for polymerization. Semi‐thin sections were prepared for orientation purposes, followed by the production of ultrathin sections, which were collected for microstructural examination. The sections were counterstained with 3% uranyl acetate and 2.7% lead citrate. Imaging was conducted using a JEM1400 transmission electron microscope. Synapses were identified by the presence of synaptic vesicles and postsynaptic densities.

### 4.13 Quantitative Real‐Time PCR (RT‐qPCR)

Total RNA was extracted using Trizol reagent (Invitrogen, cat# 15596026), and reverse transcription was conducted using the PrimeScript real time Master Mix (TAKARA, cat# RR047A). Prior to reverse transcription, genomic DNA was eliminated according to the protocol. RT‐qPCR was performed on a Bio‐Rad Connect Real‐Time PCR platform using SYBR Green Master Mix (AGbio, cat# AG11701). Relative gene expression was calculated using the 2^−ΔΔCT^ method with GAPDH as the internal control. Primer sequences are listed in supplementary Table S3.

### 4.14 Statistical Analysis

Statistical analyses were performed with Graphpad Prism (v. 9.5; GraphPad Software, La Jolla, California, USA). Unpaired Student's *t*‐test was used to determine differences between two groups. One‐way or two‐way analysis of variance (ANOVA) followed by Tukey's multiple comparisons test was used to determine differences among three or more groups as indicated in the figure legends. The statistically significant levels were set at *p* < 0.05 (*), *p* < 0.01 (**), *p* < 0.001 (***) with a confidence interval of 95%. Data were expressed as mean ± SEM. All samples or animals were included for statistical analysis unless otherwise noted in pre‐established criteria.

## Author Contributions

Conceptualization: Shifeng Xiao, Lijuan Wu, Jinwang Ye. Methodology: Shifeng Xiao, Lijuan Wu, Wenfei Qiao, Jinwang Ye. Investigation and data analysis: Lijuan Wu, Shifeng Xiao, Wenfei Qiao, Ruizhu Yue, Xing Guo, Chengjia Li, Yingling Tang. Writing preparation: Lijuan Wu, Shifeng Xiao, Jinwang Ye. Funding acquisition: Shifeng Xiao, Jinwang Ye. Research resources: Shifeng Xiao, Jinwang Ye.

## Funding

This study was supported by grants from the Natural Science Foundation of China (82101493); Guangdong Basic and Applied Basic Research Foundation (2025A1515012551); Shenzhen‐Hong Kong Institute of Brain Science‐Shenzhen Fundamental Research Institutions (Grant No. 2023SHIBS0003).

## Conflicts of Interest

The authors declare no conflicts of interest.

## Supporting information


**Figure S1:** Expression of HDAC7 in hTau transgenic mice and hTau‐overexpression primary neurons.
**Figure S2:** Effects of HDAC7 overexpression on tau acetylation in hTau‐expressing mice.
**Figure S3:** Effects of HDAC7 overexpression on CDK5, Fyn, and PP2A in primary neurons.
**Figure S4:** Effects of HDAC7 overexpression on the acetylation status of GSK3β in the absence human tau.
**Figure S5:** Validation of the ac‐GSK3β (K15) antibody.
**Figure S6:** Effects of HDAC7 pharmacological inhibition with TMP195 on GSK3β activity in hTau‐overexpressing mice.
**Figure S7:** Effects of HDAC7 catalytically inactive mutant and domain deletion mutant on tau phosphorylation in HEK293T cells.
**Figure S8:** Effects of HDAC7 deletion on GSK3β‐hTau interaction in hTau‐expressing *Hdac7* ^+/+^ and *Hdac7* ^−/−^ mice.
**Table S1:** The list of antibodies used in the study.
**Table S2:** Demographic and clinical information of human brain samples.
**Table S3:** The list of primers used for RT‐qPCR in the study.

## Data Availability

The data that support the findings of this study are available on request from the corresponding author. The data are not publicly available due to privacy or ethical restrictions.
