# Supplementary material for: HDAC7 Scaffolds GSK3β‐Tau Interaction to Drive GSK3β Activation and Tau Pathology
Source: Aging Cell. 2026 Nov 10;25(11):e70830. doi: 10.1111/acel.70830 (PMC13749147; doi:10.1111/acel.70830)
Supplement: Supplementary file 1 [file ACEL-25-e70830-s001.docx]

**Supplementary information**

**Figure S1 Expression of HDAC7 in hTau transgenic mice and hTau-overexpression primary neurons.**

(**A**) Immunofluorescence staining analysis of HDAC7 in the hippocampal CA3 region of 12-month-old WT and hTau transgenic mice, with quantification of the Manders’ M1 coefficient (HDAC7 overlapped with AT8). n = 5 mice per group. Scale bar: 80 μm. (**B** and **C**) Western blot analysis and quantification of HDAC7 in the nuclear and cytoplasmic fraction from primary cultured neurons overexpressed with hTau-GFP or control vector. n = 6 per group. Statistical significance was determined by unpaired Student’s t test. Data are shown as mean ± SEM, **p* < 0.05, ***p* < 0.01, ****p* < 0.001, n. s, not significant.

**Figure S2 Effects of HDAC7 overexpression on tau acetylation in hTau-expressing mice.**

Western blot analysis and quantification of acetylated tau (K174) in the hippocampal CA3 lysates from mice overexpressed with hTau and HDAC7. n = 6 mice per group. Statistical significance was determined by unpaired Student’s t test. Data are shown as mean ± SEM, **p* < 0.05.

**Figure S3 Effects of HDAC7 overexpression on CDK5, Fyn and PP2A in primary neurons.**

Western blot analysis and quantification of p35-p25, CDK5, Fyn, and PP2Ac in primary cultured neurons overexpressed with HDAC7 or control vector via lentivirus. n = 6 per group. Statistical significance was determined by unpaired Student’s t test. Data are shown as mean ± SEM, **p* < 0.05, ***p* < 0.01, ****p* < 0.001, n. s, not significant.

**Figure S4 Effects of HDAC7 overexpression on the acetylation status of GSK3β in the absence human tau.**

Immunoprecipitation (IP) analysis of GSK3β acetylation levels in HEK293T cells co-transfected with GSK3β-Flag and HDAC7-GFP or vector. n = 6 per group. Statistical significance was determined by unpaired Student’s t test. Data are shown as mean ± SEM, **p* < 0.05, ***p* < 0.01, ****p* < 0.001, n. s, not significant.

**Figure S5 Validation of the ac-GSK3β (K15) antibody.**

(**A**) Dot blot analysis demonstrates the reactivity of the ac-GSK3β (K15) antibody with K15-acetylated and non-acetylated peptides. (**B**) Co-IP assay analysis illustrates the reactivity of the ac-GSK3β (K15) antibody with exogenously expressed GSK3β-Flag in HEK293T cells transfected with GSK3β-Flag, GSK3β(K15R)-Flag or control vector. n = 6 per group.

**Figure S6 Effects of HDAC7 pharmacological inhibition with TMP195 on GSK3β activity in hTau-overexpressing mice.**

Western blot analysis and quantification of p-GSK3β (Tyr216) and total GSK3β in the hippocampal CA3 lysates from control and hTau-expressing mice intraperitoneally injected with TMP195. n = 6 mice per group. Statistical significance was determined by one-way ANOVA with Tukey’s post hoc analysis. Data are shown as mean ± SEM, **p* < 0.05, ***p* < 0.01, ****p* < 0.001, n. s, not significant.

**Figure S7 Effects of HDAC7 catalytically inactive mutant and domain deletion mutant on tau phosphorylation in HEK293T cells.**

(**A** and **B**) Western blot analysis and quantification of phosphorylated tau (p-Ser202, p-Thr205, p-Ser214, p-Thr217, p-Thr231, and p-Ser396) in HEK293T cells co-transfected with GSK3β-Flag, hTau-GFP, and HDAC7-HA or HDAC7(H672A)-HA or control vector. n = 6 per group. (**C** and **D**) Western blot analysis and quantification of phosphorylated tau (p-Ser202, p-Thr205, p-Ser214, p-Thr217, p-Thr231, and p-Ser396) in HEK293T cells co-transfected with GSK3β-Flag, hTau-GFP, and HDAC7-HA or N-HDAC7-HA or C-HDAC7-HA or control vector. n = 6 per group. Statistical significance was determined by one-way ANOVA with Tukey’s post hoc analysis. Data are shown as mean ± SEM, **p* < 0.05, ***p* < 0.01, ****p* < 0.001, n. s, not significant.

**Figure S8 Effects of HDAC7 deletion on GSK3β-hTau interaction in hTau-expressing *Hdac7^+/+^* and *Hdac7^-/-^* mice.**

Co-IP assay analysis of the interaction between GSK3β and hTau in hippocampal tissue lysates from hTau-expressing *Hdac7*^+/+^ and *Hdac7*^−/−^ mice. n = 4 mice per group. Statistical significance was determined by unpaired Student’s t test. Data are shown as mean ± SEM, **p* < 0.05, ***p* < 0.01, ****p* < 0.001.

**Table S1.** **The list of antibodies used in the study.**

| **Antibody** | **Catalog** | **Source** | **Dilution ratio** |
| --- | --- | --- | --- |
| Acetylated-lysine | 9441S | Cell Signaling Technology | WB: 1:1000 |
| HDAC4 | 7628S | Cell Signaling Technology | WB: 1:1000 |
| HDAC5 | 20458S | Cell Signaling Technology | WB: 1:1000 |
| HDAC7 | H2662 | Sigma-Aldrich | WB: 1:1000,IF: 1:100 |
| HDAC9 | Ab109446 | Abcam | WB: 1:1000 |
| Tau13 | 835201 | Biolegend | WB: 1:1000, IF: 1:200 |
| AT8 | MN1020 | Thermo Fisher | IF: 1:100 |
| Phospho-tau (Ser202) | 39357S | Cell Signaling Technology | WB: 1:1000 |
| Phospho-tau (Thr205) | #11108 | Sabbiotech | WB 1: 1000, IF: 1:100 |
| Phospho-tau (Ser214) | #21098 | Sabbiotech | WB 1: 1000 |
| Phospho-tau (Thr217) | #11724 | Sabbiotech | WB: 1: 1000 |
| Phospho-tau (Thr231) | #11110 | Sabbiotech | IF: 1:100 |
| Phospho-tau (Ser262) | #11111 | Sabbiotech | WB: 1:1000 |
| Phospho-tau (Ser396) | #11102 | Sabbiotech | WB: 1:1000, IF: 1:100 |
| Tau (Acetyl Lys174) | #HW181 | Sabbiotech | WB: 1:1000 |
| Beta Actin | 66009-1-Ig | ProteinTech | WB: 1:1000 |
| GSK3β | 12456S | Cell Signaling Technology | WB: 1:1000 |
| Phospho-GSK3β (Ser9) | 5558S | Cell Signaling Technology | WB: 1:1000 |
| Phospho-GSK3β (Tyr216) | 29125-1-AP | ProteinTech | WB: 1:1000, IF: 1:100 |
| Ace-GSK3β (K15) | N. A | N. A | WB: 1:500 |
| AKT | 4691S | Cell Signaling Technology | WB: 1:1000 |
| Phospho-AKT(Ser473) | 4060S | Cell Signaling Technology | WB: 1:1000 |
| Fyn | 4023S | Cell Signaling Technology | WB: 1:1000 |
| CDK5 | 14145S | Cell Signaling Technology | WB: 1:1000 |
| P35/25 | 2680S | Cell Signaling Technology | WB: 1:1000 |
| PP2Ac | 2038S | Cell Signaling Technology | WB: 1:1000 |
| Ubiquitin | MK-11-3 | MBL Life science | WB: 1:1000 |
| Synaptophysin | 4329S | Cell Signaling Technology | WB: 1:1000 |
| PSD95 | 36233S | Cell Signaling Technology | WB: 1:1000 |
| AMPA Receptor 1 (GluA1) | 13185S | Cell Signaling Technology | WB: 1:1000 |
| Synapsin-1 | 5297S | Cell Signaling Technology | WB: 1:1000 |
| DYKDDDDK Tag | 8146S | Cell Signaling Technology | WB: 1:1000, IP: 1:100 |
| HA-Tag | 3724S | Cell Signaling Technology | WB: 1:1000, IP: 1:100 |
| NeuN | OB-PGP006 | Oasis Biofarm | IF: 1:500 |
| AF488-labeled Goat Anti-Mouse IgG (H+L) | A0428 | Beyotime | IF: 1:500 |
| AF555-labeled Donkey Anti-Rabbit IgG (H+L) | A0453 | Beyotime | IF: 1:500 |
| Goat Anti-Rabbit IgG H&L (Alexa Fluor® 488) | Ab150077 | Abcam | IF: 1:500 |
| Goat Anti-Chicken IgY H&L (Alexa Fluor® 647) | Ab150175 | Abcam | IF: 1:500 |
| Donkey anti-Guinea pig IgG, AF647 | D-GP647 | Oasis Biofarm | IF: 1:1000 |

**Table S2.** **Demographic and clinical information of human brain samples.**

| **Case number** | **Gender** | **Age** | **Primary Neuropathological Diagnosis** | **Postmortem Interval (h)** |
| --- | --- | --- | --- | --- |
| 1 | Female | 86 | AD | 6.33 |
| 2 | Female | 80 | AD | 13 |
| 3 | Male | 83 | AD | 4.5 |
| 4 | Male | 97 | AD | 26.5 |
| 5 | Female | 84 | Control | 20 |
| 6 | Female | 96 | Control | 32 |
| 7 | Male | 79 | Control | 29 |
| 8 | Male | 80 | Control | 7.5 |

**Table S3.** **The list of primers used for RT-qPCR in the study.**

| **Targeted gene** | **Forward** | **Reverse** |
| --- | --- | --- |
| GAPDH (Ms) | TGTGAACGGATTTGGCCGTA | ACTGTGCCGTTGAATTTGCC |
| GAPDH (Hu) | CTGCCAACGTGTCAGTGGTG | TCAGTGTAGCCCAGGATGCC |
| HDAC2 (Ms) | GGAGGAGGCTACACAATCCG | TCTGGAGTGTTCTGGTTTGTCA |
| HDAC3 (Ms) | GCCAAGACCGTGGCGTATT | GTCCAGCTCCATAGTGGAAGT |
| HDAC4 (Ms) | AGCAGCACCAGCAGTTCCT | CCGCTTCTTCCTCCTCACTCT |
| HDAC5 (Ms) | AGCAGCACCAGCAGTTCCT | CACTCTCGCCATCCTCATCCT |
| HDAC6 (Ms) | TCCACCGGCCAAGATTCTTC | GCCTTTCTTCTTTACCTCCGCT |
| HDAC7 (Ms) | CGCAGCCAGTGTGAGTGTCT | GCTCGTTCCAGATGGTGTCAGTA |
| HDAC8 (Ms) | ACTATTGCCGGAGATCCAATGT | CCTCCTAAAATCAGAGTTGCCAG |
| HDAC9 (Ms) | GATGATGATGCCTGTGGTGGAT | TGCTGCTGCTGCTGAATAAGAA |
| HDAC10 (Ms) | GCTCCACGCACTGTCTAAG | CCTGTTAGCACAGCATCGACC |
| HDAC11 (Ms) | GTGTACTCACCACGTTACAACA | GCTCGTTGAGATAGCGCCTC |
| SIRT1 (Ms) | GCTGACGACTTCGACGACG | TCGGTCAACAGGAGGTTGTCT |
| SIRT2 (Ms) | GCCTGGGTTCCCAAAAGGAG | GAGCGGAAGTCAGGGATACC |
| SIRT3 (Ms) | ATCCCGGACTTCAGATCCCC | CAACATGAAAAAGGGCTTGGG |
| GSK3β (Ms) | TGAGGCAACGGAAAAAGGAGAT | TGACTTCCTGTGGCCTGTCA |
| GSK3β (Hu) | GGCAGCATGAAAGTTAGCAGA | GGCGACCAGTTCTCCTGAATC |
